# Supplementary material for: Ormdl3 regulation of specific ceramides is dispensable for mouse β-cell function and glucose homeostasis under obesogenic conditions
Source: Front Endocrinol (Lausanne). 2023 Apr 14;14:1170461. doi: 10.3389/fendo.2023.1170461 (PMC10140491; doi:10.3389/fendo.2023.1170461)
Supplement: Supplementary file 1 [file Table_1.docx]

**Supplementary Table 1**

| **Cpd Group** | **Cpd Name** | **ISTD?** | **Prec Ion** | **MS1 Res** | **Prod Ion** | **MS2 Res** | **Frag (V)** | **CE (V)** | **Cell Acc (V)** | **Ret Time (min)** | **Ret Window** | **Polarity** |
| --- | --- | --- | --- | --- | --- | --- | --- | --- | --- | --- | --- | --- |
| DHCeramides | Cer(d18:0/02:0) | No | 344.3 | Unit/Enh (6490) | 266.3 | Unit/Enh (6490) | 166 | 30 | 7 | 1.4 | 1 | Positive |
| DHCeramides | Cer(d18:0/02:0) | No | 344.3 | Unit/Enh (6490) | 60.3 | Unit/Enh (6490) | 166 | 28 | 7 | 1.4 | 1 | Positive |
| DHCeramides | Cer(d18:0/06:0) | No | 400.4 | Unit/Enh (6490) | 284.4 | Unit/Enh (6490) | 166 | 30 | 2 | 1.8 | 1 | Positive |
| DHCeramides | Cer(d18:0/06:0) | No | 400.4 | Unit/Enh (6490) | 266.3 | Unit/Enh (6490) | 166 | 30 | 2 | 1.8 | 1 | Positive |
| DHCeramides | Cer(d18:0/08:0) | No | 428.4 | Unit/Enh (6490) | 284.3 | Unit/Enh (6490) | 166 | 24 | 2 | 2 | 1 | Positive |
| DHCeramides | Cer(d18:0/08:0) | No | 428.4 | Unit/Enh (6490) | 266.3 | Unit/Enh (6490) | 166 | 30 | 2 | 2 | 1 | Positive |
| DHCeramides | Cer(d18:0/12:0) | No | 484.5 | Unit/Enh (6490) | 284.3 | Unit/Enh (6490) | 166 | 24 | 7 | 2.4 | 1 | Positive |
| DHCeramides | Cer(d18:0/12:0) | No | 484.5 | Unit/Enh (6490) | 266.3 | Unit/Enh (6490) | 166 | 30 | 7 | 2.4 | 1 | Positive |
| DHCeramides | Cer(d18:0/16:0) | No | 540.5 | Unit/Enh (6490) | 284.3 | Unit/Enh (6490) | 166 | 36 | 5 | 3.3 | 1 | Positive |
| DHCeramides | Cer(d18:0/16:0) | No | 540.5 | Unit/Enh (6490) | 266.3 | Unit/Enh (6490) | 166 | 30 | 4 | 3.3 | 1 | Positive |
| DHCeramides | Cer(d18:0/18:0) | No | 568.6 | Unit/Enh (6490) | 284.3 | Unit/Enh (6490) | 166 | 36 | 6 | 3.8 | 1 | Positive |
| DHCeramides | Cer(d18:0/18:0) | No | 568.6 | Unit/Enh (6490) | 266.3 | Unit/Enh (6490) | 166 | 30 | 6 | 3.8 | 1 | Positive |
| DHCeramides | Cer(d18:0/20:0) | No | 596.6 | Unit/Enh (6490) | 284.4 | Unit/Enh (6490) | 166 | 32 | 3 | 4.2 | 1 | Positive |
| DHCeramides | Cer(d18:0/20:0) | No | 596.6 | Unit/Enh (6490) | 266.3 | Unit/Enh (6490) | 166 | 30 | 3 | 4.2 | 1 | Positive |
| DHCeramides | Cer(d18:0/22:0) | No | 624.6 | Unit/Enh (6490) | 284.4 | Unit/Enh (6490) | 166 | 32 | 3 | 4.6 | 1 | Positive |
| DHCeramides | Cer(d18:0/22:0) | No | 624.6 | Unit/Enh (6490) | 266.3 | Unit/Enh (6490) | 166 | 30 | 3 | 4.6 | 1 | Positive |
| DHCeramides | Cer(d18:0/24:0) | No | 652.7 | Unit/Enh (6490) | 284.4 | Unit/Enh (6490) | 166 | 32 | 3 | 5.5 | 1 | Positive |
| DHCeramides | Cer(d18:0/24:0) | No | 652.7 | Unit/Enh (6490) | 266.3 | Unit/Enh (6490) | 166 | 30 | 3 | 5.5 | 1 | Positive |
| DHCeramides | Cer(d18:0/24:1) | No | 650.7 | Unit/Enh (6490) | 284.3 | Unit/Enh (6490) | 166 | 28 | 3 | 5.2 | 1 | Positive |
| DHCeramides | Cer(d18:0/24:1) | No | 650.7 | Unit/Enh (6490) | 266.3 | Unit/Enh (6490) | 166 | 30 | 3 | 5.2 | 1 | Positive |
| Ceramides | Cer(d18:1/02:0) | No | 342.3 | Unit/Enh (6490) | 264.3 | Unit/Enh (6490) | 166 | 30 | 7 | 1.4 | 1 | Positive |
| Ceramides | Cer(d18:1/02:0) | No | 342.3 | Unit/Enh (6490) | 252.3 | Unit/Enh (6490) | 166 | 30 | 7 | 1.4 | 1 | Positive |
| Ceramides | Cer(d18:1/12:0) | No | 464.5 | Unit/Enh (6490) | 282.3 | Unit/Enh (6490) | 166 | 30 | 4 | 2 | 1 | Positive |
| Ceramides | Cer(d18:1/12:0) | No | 464.5 | Unit/Enh (6490) | 264.2 | Unit/Enh (6490) | 166 | 30 | 4 | 2 | 1 | Positive |
| Ceramides | Cer(d18:1/14:0) | No | 492.5 | Unit/Enh (6490) | 282.3 | Unit/Enh (6490) | 166 | 30 | 4 | 2.7 | 1 | Positive |
| Ceramides | Cer(d18:1/14:0) | No | 492.5 | Unit/Enh (6490) | 264.2 | Unit/Enh (6490) | 166 | 30 | 4 | 2.7 | 1 | Positive |
| Ceramides | Cer(d18:1/16:0) | No | 520.5 | Unit/Enh (6490) | 282.3 | Unit/Enh (6490) | 166 | 30 | 4 | 3.4 | 1 | Positive |
| Ceramides | Cer(d18:1/16:0) | No | 520.5 | Unit/Enh (6490) | 264.2 | Unit/Enh (6490) | 166 | 30 | 4 | 3.4 | 1 | Positive |
| Ceramides | Cer(d18:1/16:1) | No | 518.5 | Unit/Enh (6490) | 282.3 | Unit/Enh (6490) | 166 | 30 | 4 | 2.7 | 1 | Positive |
| Ceramides | Cer(d18:1/16:1) | No | 518.5 | Unit/Enh (6490) | 264.2 | Unit/Enh (6490) | 166 | 30 | 4 | 2.7 | 1 | Positive |
| Ceramides | Cer(d18:1/17:0) | No | 534.5 | Unit/Enh (6490) | 282.3 | Unit/Enh (6490) | 166 | 30 | 4 | 3.5 | 1 | Positive |
| Ceramides | Cer(d18:1/17:0) | No | 534.5 | Unit/Enh (6490) | 264.2 | Unit/Enh (6490) | 166 | 30 | 4 | 3.5 | 1 | Positive |
| Ceramides | Cer(d18:1/18:0) | No | 548.5 | Unit/Enh (6490) | 282.3 | Unit/Enh (6490) | 166 | 30 | 4 | 3.8 | 1 | Positive |
| Ceramides | Cer(d18:1/18:0) | No | 548.5 | Unit/Enh (6490) | 264.2 | Unit/Enh (6490) | 166 | 30 | 4 | 3.8 | 1 | Positive |
| Ceramides | Cer(d18:1/18:1) | No | 546.5 | Unit/Enh (6490) | 282.3 | Unit/Enh (6490) | 166 | 30 | 4 | 3.4 | 1 | Positive |
| Ceramides | Cer(d18:1/18:1) | No | 546.5 | Unit/Enh (6490) | 264.2 | Unit/Enh (6490) | 166 | 30 | 4 | 3.4 | 1 | Positive |
| Ceramides | Cer(d18:1/19:0) | No | 562.6 | Unit/Enh (6490) | 282.3 | Unit/Enh (6490) | 166 | 30 | 4 | 3.4 | 1 | Positive |
| Ceramides | Cer(d18:1/19:0) | No | 562.6 | Unit/Enh (6490) | 264.2 | Unit/Enh (6490) | 166 | 30 | 4 | 3.4 | 1 | Positive |
| Ceramides | Cer(d18:1/20:0) | No | 576.6 | Unit/Enh (6490) | 282.3 | Unit/Enh (6490) | 166 | 30 | 4 | 4.3 | 1 | Positive |
| Ceramides | Cer(d18:1/20:0) | No | 576.6 | Unit/Enh (6490) | 264.2 | Unit/Enh (6490) | 166 | 30 | 4 | 4.3 | 1 | Positive |
| Ceramides | Cer(d18:1/22:0) | No | 604.6 | Unit/Enh (6490) | 282.3 | Unit/Enh (6490) | 166 | 30 | 4 | 4.7 | 1 | Positive |
| Ceramides | Cer(d18:1/22:0) | No | 604.6 | Unit/Enh (6490) | 264.2 | Unit/Enh (6490) | 166 | 30 | 4 | 4.7 | 1 | Positive |
| Ceramides | Cer(d18:1/22:1) | No | 602.6 | Unit/Enh (6490) | 282.3 | Unit/Enh (6490) | 166 | 30 | 4 | 4.2 | 1 | Positive |
| Ceramides | Cer(d18:1/22:1) | No | 602.6 | Unit/Enh (6490) | 264.2 | Unit/Enh (6490) | 166 | 30 | 4 | 4.2 | 1 | Positive |
| Ceramides | Cer(d18:1/23:0) | No | 618.6 | Unit/Enh (6490) | 282.3 | Unit/Enh (6490) | 166 | 30 | 5 | 4.9 | 1 | Positive |
| Ceramides | Cer(d18:1/23:0) | No | 618.6 | Unit/Enh (6490) | 264.2 | Unit/Enh (6490) | 166 | 30 | 4 | 4.9 | 1 | Positive |
| Ceramides | Cer(d18:1/24:0) | No | 632.6 | Unit/Enh (6490) | 282.3 | Unit/Enh (6490) | 166 | 30 | 5 | 5.2 | 1 | Positive |
| Ceramides | Cer(d18:1/24:0) | No | 632.6 | Unit/Enh (6490) | 264.2 | Unit/Enh (6490) | 166 | 30 | 4 | 5.2 | 1 | Positive |
| Ceramides | Cer(d18:1/24:1) | No | 630.6 | Unit/Enh (6490) | 282.3 | Unit/Enh (6490) | 166 | 30 | 4 | 4.7 | 1 | Positive |
| Ceramides | Cer(d18:1/24:1) | No | 630.6 | Unit/Enh (6490) | 264.2 | Unit/Enh (6490) | 166 | 30 | 4 | 4.7 | 1 | Positive |
| Ceramides | Cer(d18:1/26:0) | No | 660.7 | Unit/Enh (6490) | 282.3 | Unit/Enh (6490) | 166 | 30 | 4 | 5.8 | 1 | Positive |
| Ceramides | Cer(d18:1/26:0) | No | 660.7 | Unit/Enh (6490) | 264.2 | Unit/Enh (6490) | 166 | 30 | 4 | 5.8 | 1 | Positive |
| Ceramides | Cer(d18:1/26:1) | No | 658.7 | Unit/Enh (6490) | 282.3 | Unit/Enh (6490) | 166 | 30 | 4 | 5.2 | 1 | Positive |
| Ceramides | Cer(d18:1/26:1) | No | 658.7 | Unit/Enh (6490) | 264.2 | Unit/Enh (6490) | 166 | 30 | 5 | 5.2 | 1 | Positive |
| IS | Cer(d18:1/8:0) | Yes | 426.688 | Unit/Enh (6490) | 282.3 | Unit/Enh (6490) | 166 | 30 | 4 | 2.8 | 1 | Positive |
| IS | Cer(d18:1/8:0) | Yes | 426.688 | Unit/Enh (6490) | 264.2 | Unit/Enh (6490) | 166 | 30 | 4 | 2.8 | 1 | Positive |
| IS | Cer(d18:1d7/15:0) | Yes | 530.917 | Unit/Enh (6490) | 271.3 | Unit/Enh (6490) | 166 | 30 | 4 | 4.1 | 1 | Positive |
| IS | Cer(d18:1d7/16:0) | Yes | 527.5 | Unit/Enh (6490) | 271.3 | Unit/Enh (6490) | 166 | 30 | 4 | 4.3 | 1 | Positive |
| IS | Cer(d18:1d7/18:0) | Yes | 555.5 | Unit/Enh (6490) | 271.3 | Unit/Enh (6490) | 166 | 30 | 4 | 4.8 | 1 | Positive |
| IS | Cer(d18:1d7/24:0) | Yes | 639.6 | Unit/Enh (6490) | 271.3 | Unit/Enh (6490) | 166 | 30 | 4 | 6.4 | 1 | Positive |
| IS | Cer(d18:1d7/24:1) | Yes | 637.6 | Unit/Enh (6490) | 271.3 | Unit/Enh (6490) | 166 | 30 | 4 | 5.8 | 1 | Positive |
| Ceramides | Cer(d18:2/16:0) | No | 536.5 | Unit/Enh (6490) | 280.3 | Unit/Enh (6490) | 166 | 20 | 4 | 3.1 | 1 | Positive |
| Ceramides | Cer(d18:2/16:0) | No | 536.5 | Unit/Enh (6490) | 262.2 | Unit/Enh (6490) | 166 | 20 | 4 | 3.1 | 1 | Positive |
| Ceramides | Cer(d18:2/18:0) | No | 564.5 | Unit/Enh (6490) | 280.3 | Unit/Enh (6490) | 166 | 28 | 4 | 3.6 | 1 | Positive |
| Ceramides | Cer(d18:2/18:0) | No | 564.5 | Unit/Enh (6490) | 262.3 | Unit/Enh (6490) | 166 | 28 | 4 | 3.6 | 1 | Positive |
| Ceramides | Cer(d18:2/20:0) | No | 592.6 | Unit/Enh (6490) | 280.3 | Unit/Enh (6490) | 166 | 28 | 4 | 4.2 | 1 | Positive |
| Ceramides | Cer(d18:2/20:0) | No | 592.6 | Unit/Enh (6490) | 262.3 | Unit/Enh (6490) | 166 | 28 | 4 | 4.2 | 1 | Positive |
| Ceramides | Cer(d18:2/22:0) | No | 620.6 | Unit/Enh (6490) | 280.3 | Unit/Enh (6490) | 166 | 28 | 4 | 5.2 | 1 | Positive |
| Ceramides | Cer(d18:2/22:0) | No | 620.6 | Unit/Enh (6490) | 262.3 | Unit/Enh (6490) | 166 | 28 | 4 | 5.2 | 1 | Positive |
| Ceramides | Cer(d18:2/24:0) | No | 648.6 | Unit/Enh (6490) | 280.3 | Unit/Enh (6490) | 166 | 28 | 4 | 5.7 | 1 | Positive |
| Ceramides | Cer(d18:2/24:0) | No | 648.6 | Unit/Enh (6490) | 262.3 | Unit/Enh (6490) | 166 | 28 | 4 | 5.7 | 1 | Positive |
| Ceramides | Cer(d18:2/24:1) | No | 646.6 | Unit/Enh (6490) | 280.3 | Unit/Enh (6490) | 166 | 28 | 4 | 5 | 1 | Positive |
| Ceramides | Cer(d18:2/24:1) | No | 646.6 | Unit/Enh (6490) | 262.3 | Unit/Enh (6490) | 166 | 28 | 4 | 5 | 1 | Positive |
| deoxyCer | Cer(m18:1/16:0) | No | 522.5 | Unit/Enh (6490) | 266.3 | Unit/Enh (6490) | 166 | 30 | 4 | 3.4 | 1 | Positive |
| deoxyCer | Cer(m18:1/18:0) | No | 550.6 | Unit/Enh (6490) | 266.3 | Unit/Enh (6490) | 166 | 30 | 4 | 3.8 | 1 | Positive |
| deoxyCer | Cer(m18:1/20:0) | No | 578.6 | Unit/Enh (6490) | 266.3 | Unit/Enh (6490) | 166 | 30 | 4 | 4 | 1 | Positive |
| deoxyCer | Cer(m18:1/22:0) | No | 606.6 | Unit/Enh (6490) | 266.3 | Unit/Enh (6490) | 166 | 30 | 4 | 5 | 1 | Positive |
| deoxyCer | Cer(m18:1/24:0) | No | 634.6 | Unit/Enh (6490) | 266.3 | Unit/Enh (6490) | 166 | 30 | 4 | 5.4 | 1 | Positive |
| deoxyCer | Cer(m18:1/24:1) | No | 632.6 | Unit/Enh (6490) | 266.3 | Unit/Enh (6490) | 166 | 30 | 4 | 4.9 | 1 | Positive |
| PhytoCeramides | Cer(t18:0/16:0) | No | 556.5 | Unit/Enh (6490) | 300.3 | Unit/Enh (6490) | 166 | 24 | 4 | 2.6 | 1 | Positive |
| Phytoceramides | Cer(t18:0/16:0) | No | 556.5 | Unit/Enh (6490) | 282.3 | Unit/Enh (6490) | 166 | 24 | 4 | 2.6 | 1 | Positive |
| Phytoceramides | Cer(t18:0/18:0) | No | 584.6 | Unit/Enh (6490) | 300.3 | Unit/Enh (6490) | 166 | 24 | 4 | 3.3 | 1 | Positive |
| Phytoceramides | Cer(t18:0/18:0) | No | 584.6 | Unit/Enh (6490) | 282.4 | Unit/Enh (6490) | 166 | 28 | 4 | 3.3 | 1 | Positive |
| Phytoceramides | Cer(t18:0/20:0) | No | 612.6 | Unit/Enh (6490) | 300.3 | Unit/Enh (6490) | 166 | 24 | 4 | 3.7 | 1 | Positive |
| Phytoceramides | Cer(t18:0/20:0) | No | 612.6 | Unit/Enh (6490) | 282.4 | Unit/Enh (6490) | 166 | 28 | 4 | 3.7 | 1 | Positive |
| Phytoceramides | Cer(t18:0/22:0) | No | 640.6 | Unit/Enh (6490) | 300.3 | Unit/Enh (6490) | 166 | 24 | 4 | 3.7 | 1 | Positive |
| Phytoceramides | Cer(t18:0/22:0) | No | 640.6 | Unit/Enh (6490) | 282.4 | Unit/Enh (6490) | 166 | 28 | 4 | 3.7 | 1 | Positive |
| Phytoceramides | Cer(t18:0/24:0) | No | 668.7 | Unit/Enh (6490) | 300.3 | Unit/Enh (6490) | 166 | 24 | 4 | 4.2 | 1 | Positive |
| Phytoceramides | Cer(t18:0/24:0) | No | 668.7 | Unit/Enh (6490) | 282.4 | Unit/Enh (6490) | 166 | 28 | 4 | 4.2 | 1 | Positive |
| Phytoceramides | Cer(t18:0/24:1) | No | 666.6 | Unit/Enh (6490) | 300.3 | Unit/Enh (6490) | 166 | 24 | 4 | 4 | 1 | Positive |
| Phytoceramides | Cer(t18:0/24:1) | No | 666.6 | Unit/Enh (6490) | 282.4 | Unit/Enh (6490) | 166 | 28 | 4 | 4 | 1 | Positive |
| IS | Cerd18:0/18:1 | Yes | 566.5 | Unit/Enh (6490) | 284.3 | Unit/Enh (6490) | 166 | 32 | 5 | 4.5 | 1 | Positive |
| IS | Cerd18:0/18:1 | Yes | 566.5 | Unit/Enh (6490) | 266.3 | Unit/Enh (6490) | 166 | 32 | 5 | 4.5 | 1 | Positive |
| DHGSC | GlcCer (d18:0/16:0) | No | 702.6 | Unit/Enh (6490) | 284.3 | Unit/Enh (6490) | 166 | 29 | 4 | 3.5 | 1 | Positive |
| DHGSC | GlcCer (d18:0/16:0) | No | 702.6 | Unit/Enh (6490) | 266.3 | Unit/Enh (6490) | 166 | 29 | 4 | 3.5 | 1 | Positive |
| DHGSC | GlcCer (d18:0/18:0) | No | 730.6 | Unit/Enh (6490) | 284.3 | Unit/Enh (6490) | 166 | 29 | 4 | 3.3 | 1 | Positive |
| DHGSC | GlcCer (d18:0/18:0) | No | 730.6 | Unit/Enh (6490) | 266.3 | Unit/Enh (6490) | 166 | 29 | 4 | 3.3 | 1 | Positive |
| GSC | GlcCer (d18:0/2:0) | No | 506.4 | Unit/Enh (6490) | 266.3 | Unit/Enh (6490) | 166 | 29 | 4 | 2 | 1 | Positive |
| DHGSC | GlcCer (d18:0/20:0) | No | 758.6 | Unit/Enh (6490) | 284.3 | Unit/Enh (6490) | 166 | 29 | 4 | 4.3 | 1 | Positive |
| DHGSC | GlcCer (d18:0/20:0) | No | 758.6 | Unit/Enh (6490) | 266.3 | Unit/Enh (6490) | 166 | 29 | 4 | 4.3 | 1 | Positive |
| DHGSC | GlcCer (d18:0/22:0) | No | 786.7 | Unit/Enh (6490) | 284.3 | Unit/Enh (6490) | 166 | 29 | 4 | 4.7 | 1 | Positive |
| DHGSC | GlcCer (d18:0/22:0) | No | 786.7 | Unit/Enh (6490) | 266.3 | Unit/Enh (6490) | 166 | 29 | 4 | 4.7 | 1 | Positive |
| DHGSC | GlcCer (d18:0/24:0) | No | 814.7 | Unit/Enh (6490) | 284.3 | Unit/Enh (6490) | 166 | 29 | 4 | 5 | 1 | Positive |
| DHGSC | GlcCer (d18:0/24:0) | No | 814.7 | Unit/Enh (6490) | 266.3 | Unit/Enh (6490) | 166 | 29 | 4 | 5 | 1 | Positive |
| DHGSC | GlcCer (d18:0/24:1) | No | 812.7 | Unit/Enh (6490) | 284.3 | Unit/Enh (6490) | 166 | 29 | 4 | 4.9 | 1 | Positive |
| DHGSC | GlcCer (d18:0/24:1) | No | 812.7 | Unit/Enh (6490) | 266.3 | Unit/Enh (6490) | 166 | 29 | 4 | 4.9 | 1 | Positive |
| GSC | GlcCer (d18:1/16:0) | No | 700.6 | Unit/Enh (6490) | 282.3 | Unit/Enh (6490) | 166 | 29 | 4 | 3.4 | 1 | Positive |
| GSC | GlcCer (d18:1/16:0) | No | 700.6 | Unit/Enh (6490) | 264.3 | Unit/Enh (6490) | 166 | 29 | 4 | 3.4 | 1 | Positive |
| GSC | GlcCer (d18:1/18:0) | No | 728.6 | Unit/Enh (6490) | 282.3 | Unit/Enh (6490) | 166 | 29 | 4 | 3.8 | 1 | Positive |
| GSC | GlcCer (d18:1/18:0) | No | 728.6 | Unit/Enh (6490) | 264.2 | Unit/Enh (6490) | 166 | 29 | 4 | 3.8 | 1 | Positive |
| GSC | GlcCer (d18:1/2:0) | No | 504.4 | Unit/Enh (6490) | 264.3 | Unit/Enh (6490) | 166 | 29 | 4 | 2.1 | 1 | Positive |
| GSC | GlcCer (d18:1/2:0) | No | 504.4 | Unit/Enh (6490) | 174.1 | Unit/Enh (6490) | 166 | 29 | 4 | 2.1 | 1 | Positive |
| GSC | GlcCer (d18:1/20:0) | No | 756.6 | Unit/Enh (6490) | 282.3 | Unit/Enh (6490) | 166 | 29 | 4 | 4.2 | 1 | Positive |
| GSC | GlcCer (d18:1/20:0) | No | 756.6 | Unit/Enh (6490) | 264.2 | Unit/Enh (6490) | 166 | 29 | 4 | 4.2 | 1 | Positive |
| GSC | GlcCer (d18:1/22:0) | No | 784.7 | Unit/Enh (6490) | 282.3 | Unit/Enh (6490) | 166 | 29 | 4 | 4.7 | 1 | Positive |
| GSC | GlcCer (d18:1/22:0) | No | 784.7 | Unit/Enh (6490) | 264.2 | Unit/Enh (6490) | 166 | 29 | 4 | 4.7 | 1 | Positive |
| GSC | GlcCer (d18:1/24:0) | No | 812.7 | Unit/Enh (6490) | 282.3 | Unit/Enh (6490) | 166 | 29 | 4 | 4.6 | 1 | Positive |
| GSC | GlcCer (d18:1/24:0) | No | 812.7 | Unit/Enh (6490) | 264.2 | Unit/Enh (6490) | 166 | 29 | 4 | 4.6 | 1 | Positive |
| GSC | GlcCer (d18:1/24:1) | No | 810.7 | Unit/Enh (6490) | 282.3 | Unit/Enh (6490) | 166 | 29 | 4 | 4.7 | 1 | Positive |
| GSC | GlcCer (d18:1/24:1) | No | 810.7 | Unit/Enh (6490) | 264.2 | Unit/Enh (6490) | 166 | 29 | 4 | 4.7 | 1 | Positive |
| IS | GlcCer (d18:1/8.0) | Yes | 589.44 | Unit/Enh (6490) | 282.3 | Unit/Enh (6490) | 166 | 29 | 4 | 2.6 | 1 | Positive |
| IS | GlcCer (d18:1/8.0) | Yes | 589.44 | Unit/Enh (6490) | 264.3 | Unit/Enh (6490) | 166 | 29 | 4 | 2.6 | 1 | Positive |
| Sphingolipids | S1P | No | 380.3 | Unit/Enh (6490) | 264.2 | Unit/Enh (6490) | 166 | 12 | 4 | 2.6 | 1 | Positive |
| Sphingolipids | S1P | No | 380.3 | Unit/Enh (6490) | 247.2 | Unit/Enh (6490) | 166 | 12 | 4 | 2.6 | 1 | Positive |
| Sphingolipids | S1P | No | 380.3 | Unit/Enh (6490) | 82.1 | Unit/Enh (6490) | 166 | 37 | 4 | 2.6 | 1 | Positive |
| Sphingolipids | Sa1P | No | 382.3 | Unit/Enh (6490) | 284.3 | Unit/Enh (6490) | 166 | 15 | 4 | 3.6 | 1 | Positive |
| Sphingolipids | Sa1P | No | 382.3 | Unit/Enh (6490) | 266.2 | Unit/Enh (6490) | 166 | 20 | 4 | 3.6 | 1 | Positive |
| Sphingolipids | Sa1P | No | 382.3 | Unit/Enh (6490) | 249.2 | Unit/Enh (6490) | 166 | 37 | 4 | 3.6 | 1 | Positive |
| DHSM | SM(d18:0/16:0) | No | 705.6 | Unit/Enh (6490) | 266.3 | Unit/Enh (6490) | 166 | 29 | 4 | 2.6 | 1 | Positive |
| DHSM | SM(d18:0/16:0) | No | 705.6 | Unit/Enh (6490) | 184.4 | Unit/Enh (6490) | 166 | 29 | 4 | 2.6 | 1 | Positive |
| DHSM | SM(d18:0/18:0) | No | 733.6 | Unit/Enh (6490) | 266.3 | Unit/Enh (6490) | 166 | 29 | 4 | 2.6 | 1 | Positive |
| DHSM | SM(d18:0/18:0) | No | 733.6 | Unit/Enh (6490) | 184.4 | Unit/Enh (6490) | 166 | 29 | 4 | 2.6 | 1 | Positive |
| DHSM | SM(d18:0/2:0) | No | 509.4 | Unit/Enh (6490) | 184.1 | Unit/Enh (6490) | 166 | 29 | 4 | 1.7 | 1 | Positive |
| DHSM | SM(d18:0/20:0) | No | 761.6 | Unit/Enh (6490) | 266.3 | Unit/Enh (6490) | 166 | 29 | 4 | 3.9 | 1 | Positive |
| DHSM | SM(d18:0/20:0) | No | 761.6 | Unit/Enh (6490) | 184.4 | Unit/Enh (6490) | 166 | 29 | 4 | 3.9 | 1 | Positive |
| DHSM | SM(d18:0/22:0) | No | 789.7 | Unit/Enh (6490) | 266.3 | Unit/Enh (6490) | 166 | 29 | 4 | 4.4 | 1 | Positive |
| DHSM | SM(d18:0/22:0) | No | 789.7 | Unit/Enh (6490) | 184.4 | Unit/Enh (6490) | 166 | 29 | 4 | 4.4 | 1 | Positive |
| DHSM | SM(d18:0/24:0) | No | 817.7 | Unit/Enh (6490) | 266.3 | Unit/Enh (6490) | 166 | 29 | 4 | 4.8 | 1 | Positive |
| DHSM | SM(d18:0/24:0) | No | 817.7 | Unit/Enh (6490) | 184.4 | Unit/Enh (6490) | 166 | 29 | 4 | 4.8 | 1 | Positive |
| DHSM | SM(d18:0/24:1) | No | 815.7 | Unit/Enh (6490) | 266.3 | Unit/Enh (6490) | 166 | 29 | 4 | 4.4 | 1 | Positive |
| DHSM | SM(d18:0/24:1) | No | 815.7 | Unit/Enh (6490) | 184.4 | Unit/Enh (6490) | 166 | 29 | 4 | 4.4 | 1 | Positive |
| SM | SM(d18:1/14:0) | No | 675.5 | Unit/Enh (6490) | 264.3 | Unit/Enh (6490) | 166 | 29 | 4 | 2.3 | 1 | Positive |
| SM | SM(d18:1/14:0) | No | 675.5 | Unit/Enh (6490) | 184.4 | Unit/Enh (6490) | 166 | 29 | 4 | 2.3 | 1 | Positive |
| SM | SM(d18:1/16:0) | No | 703.6 | Unit/Enh (6490) | 264.3 | Unit/Enh (6490) | 166 | 29 | 4 | 2.5 | 1 | Positive |
| SM | SM(d18:1/16:0) | No | 703.6 | Unit/Enh (6490) | 184.4 | Unit/Enh (6490) | 166 | 29 | 4 | 2.5 | 1 | Positive |
| IS | SM(d18:1/17:0) | Yes | 717.6 | Unit/Enh (6490) | 264.3 | Unit/Enh (6490) | 166 | 29 | 4 | 3 | 1 | Positive |
| IS | SM(d18:1/17:0) | Yes | 717.6 | Unit/Enh (6490) | 184.4 | Unit/Enh (6490) | 166 | 29 | 4 | 3 | 1 | Positive |
| SM | SM(d18:1/18:0) | No | 731.6 | Unit/Enh (6490) | 264.3 | Unit/Enh (6490) | 166 | 29 | 4 | 2.9 | 1 | Positive |
| SM | SM(d18:1/18:0) | No | 731.6 | Unit/Enh (6490) | 184.4 | Unit/Enh (6490) | 166 | 29 | 4 | 2.9 | 1 | Positive |
| IS | SM(d18:1/18:1)d9 | Yes | 738.6 | Unit/Enh (6490) | 264.3 | Unit/Enh (6490) | 166 | 29 | 4 | 3.9 | 1 | Positive |
| IS | SM(d18:1/18:1)d9 | Yes | 738.6 | Unit/Enh (6490) | 184.1 | Unit/Enh (6490) | 166 | 29 | 4 | 3.9 | 1 | Positive |
| SM | SM(d18:1/2:0) | No | 507.4 | Unit/Enh (6490) | 184.1 | Unit/Enh (6490) | 166 | 29 | 4 | 2 | 1 | Positive |
| SM | SM(d18:1/20:0) | No | 759.6 | Unit/Enh (6490) | 264.3 | Unit/Enh (6490) | 166 | 29 | 4 | 3.8 | 1 | Positive |
| SM | SM(d18:1/20:0) | No | 759.6 | Unit/Enh (6490) | 184.4 | Unit/Enh (6490) | 166 | 29 | 4 | 3.8 | 1 | Positive |
| SM | SM(d18:1/22:0) | No | 787.7 | Unit/Enh (6490) | 264.3 | Unit/Enh (6490) | 166 | 29 | 4 | 3.9 | 1 | Positive |
| SM | SM(d18:1/22:0) | No | 787.7 | Unit/Enh (6490) | 184.4 | Unit/Enh (6490) | 166 | 29 | 4 | 3.9 | 1 | Positive |
| SM | SM(d18:1/24:0) | No | 815.7 | Unit/Enh (6490) | 264.3 | Unit/Enh (6490) | 166 | 29 | 4 | 4.5 | 1 | Positive |
| SM | SM(d18:1/24:0) | No | 815.7 | Unit/Enh (6490) | 184.4 | Unit/Enh (6490) | 166 | 29 | 4 | 4.5 | 1 | Positive |
| SM | SM(d18:1/24:1) | No | 813.7 | Unit/Enh (6490) | 264.3 | Unit/Enh (6490) | 166 | 29 | 4 | 4 | 1 | Positive |
| SM | SM(d18:1/24:1) | No | 813.7 | Unit/Enh (6490) | 184.4 | Unit/Enh (6490) | 166 | 29 | 4 | 4 | 1 | Positive |
| Sphinganine | Sphinganine | No | 302.3 | Unit/Enh (6490) | 284.2 | Unit/Enh (6490) | 166 | 8 | 7 | 1.1 | 1 | Positive |
| Sphinganine | Sphinganine | No | 302.3 | Unit/Enh (6490) | 254.3 | Unit/Enh (6490) | 166 | 20 | 7 | 1.1 | 1 | Positive |
| Sphingosine | Sphingosine | No | 300.3 | Unit/Enh (6490) | 282.3 | Unit/Enh (6490) | 166 | 8 | 6 | 1 | 1 | Positive |
| Sphingosine | Sphingosine | No | 300.3 | Unit/Enh (6490) | 252.3 | Unit/Enh (6490) | 166 | 20 | 6 | 1 | 1 | Positive |
